# Acceptability of the Brushing RemInder 4 Good oral HealTh (BRIGHT) trial intervention: a qualitative study of perspectives of young people and school staff

**DOI:** 10.1186/s12903-022-02073-w

**Published:** 2022-02-23

**Authors:** Sarab Elyousfi, Nicola Innes, Heather Leggett, Hannah Ainsworth, Ivor G. Chestnutt, Peter Day, Mark Robertson, Sue Pavitt, Ian Kellar, Donna Dey, Zoe Marshman

**Affiliations:** 1 School of Clinical Dentistry, University of Sheffield, Claremont Crescent, Sheffield, S10 2TA UK; 2 School of Dentistry, Cardiff University, Heath Park, Cardiff, CF14 4XY UK; 3 Department of Health Sciences, University of York, York, YO10 5DD UK; 4 School of Dentistry, University of Leeds, Leeds, LS2 9LU UK; 5 Bradford Community Dental Service, Bradford District Care NHS Foundation Trust, Bradford, UK; 6 School of Dentistry, University of Dundee, Park Place, Dundee, DD6 8EF UK; 7 School of Psychology, University of Leeds, Lifton Place, Leeds, LS2 9JT UK; 8 School of Education and Social Work, University of Dundee, Nethergate, Dundee, DD1 4HN UK

**Keywords:** Oral health, Behaviour change, Text messages, Mhealth, Young people

## Abstract

**Background:**

The Brushing RemInder 4 Good oral HealTh (BRIGHT) trial is investigating the clinical and cost-effectiveness of a multi-component behaviour change intervention to reduce the prevalence of dental caries in young people from deprived areas aged 11–13 years. Mobile health has gained popularity in delivering behaviour change interventions for improving oral health. The intervention, based on behaviour change theory, consists of two components; a single classroom-based session embedded in the school curriculum and a series of follow-up text messages (SMS) delivered twice daily to participants. This element of the process evaluation aimed to explore the acceptability of the BRIGHT intervention for pupils and school staff.

**Methods:**

Qualitative study, based on the concept of acceptability. Focus groups were conducted with 50 pupils, from six secondary schools across the UK, who had received the intervention. Semi-structured interviews were conducted with 12 members of staff. Purposive maximum variation sampling was used. Interviews were transcribed verbatim and analysed using a framework approach.

**Results:**

In line with the theoretical framework of acceptability, affective attitude, perceived effectiveness, ethicality, burden and self-efficacy were identified as factors that affect the acceptability of the BRIGHT intervention. Pupil participants appreciated learning about the consequences of inadequate brushing particularly the photographs of carious teeth during the classroom-based session. More detailed information on brushing techniques and follow-up lessons on oral health were recommended by pupils. In terms of the SMS, the data suggest that pupil participants found them to be helpful reminders for brushing their teeth. To further improve acceptability, more choice over the timing of the messages and greater interactivity to reduce tedium were suggested. Staff participants recognised the value of the lesson and reported that in general the content was suitable for their pupils. Having the lesson material prepared for them, having the necessary support and whether it was included in the curriculum, were factors that improved acceptability.

**Conclusion:**

Overall, pupils and staff found the BRIGHT intervention acceptable and made some suggestions which could be adopted in any subsequent implementation of the intervention.

**Supplementary Information:**

The online version contains supplementary material available at 10.1186/s12903-022-02073-w.

## Background

Dental caries remains highly prevalent and its distribution reflects social inequalities [1]. Adolescence is a time of transitions, with changes in lifestyle which can be associated with increased risk of dental caries due to irregular tooth brushing, insufficient fluoride exposure, and an increase in consumption of sugar-sweetened drinks [2, 3]. Furthermore, independent health practices that develop during adolescence continue into adulthood [4]. Within the UK, oral health promotion initiatives predominantly target children under the age of 11 years [5] and few interventions focus on improving the oral health of those in secondary school [6]. Existing interventions have predominantly involved oral health education only, without being underpinned by behaviour change theory or embedded within the school curriculum as recommended by the World Health Organisation’s Health Promoting Schools framework [7].

Mobile phone short-message service (SMS) messages have been gaining popularity in delivering behaviour change interventions in dentistry [8–10]. In contrast to the medical field, however little research has been conducted about the potential for SMS interventions to improve oral health with a particular paucity of randomised controlled trials [7–9].To address this the Brushing RemInder 4 Good oral HealTh (BRIGHT) trial is investigating the clinical and cost-effectiveness of improving the oral health of young people, aged 11–13 years living in deprived areas, through increased frequency of tooth brushing with a fluoride toothpaste [11]. It is a complex behaviour change intervention; consisting of a classroom-based lesson embedded in the school curriculum, followed by a series of text messages delivered twice daily to individual pupils’ own mobile phones.

The lesson was developed to be appropriate for the curricula as part of Personal, Social, Health and Economic Education (PSHE) in England and Wales and Health and Wellbeing in Scotland. Text messages were co-designed with young people who helped develop the content to be suitable for the age group. They were then piloted for two weeks with young people who suggested offering choice for the timings of the messages and an option for pupils to stop the messages and restart them at any time [12].

A mixed-method process evaluation was embedded in the BRIGHT trial at the funding application stage and developed alongside the protocol development. The aim was to inform any subsequent implementation and understand how and why the intervention was effective or ineffective and to contribute to the interpretation of the results of the outcome evaluation. The process evaluation has involved both qualitative and quantitative components. A key part of the process evaluation was assessing the acceptability of the intervention, as interventions cannot be effective if they are not accepted by the participants. Thus, acceptability is a prerequisite but not sufficient for the success of an intervention [13]. Acceptability reflects the extent to which the intervention is considered appropriate according to anticipated or experienced emotional and cognitive reactions to the intervention [13].

The aim of this qualitative study was to explore the acceptability of the classroom-based lesson from the perspectives of pupils and school staff and the text messages from the perspectives of pupils who received them. This qualitative study formed one component of the process evaluation of the BRIGHT trial.

## Method

This qualitative study included focus group interviews with pupils from the intervention arm of the BRIGHT trial and semi-structured interviews with members of school staff involved with the intervention. An updated version of the BRIGHT trial protocol is available at https://www.fundingawards.nihr.ac.uk/award/15/166/08. The East of Scotland Research Ethics Committee provided ethical approval for the trial, including the qualitative study (REC reference: 17/ES/0096), and all methods were performed in accordance with their guidelines. The consolidated criteria for reporting qualitative research (COREQ) guidelines were followed [14] (Additional file 3).

As part of the BRIGHT trial, a youth forum was established with the Children and Young People’s Empowerment Project (Chilypep), a youth enablement charity based in South Yorkshire. The youth forum was involved throughout the trial to advise on protocol development, participant recruitment, to optimise continued engagement with hard-to-reach pupils, to facilitate focus group discussions and aid in the interpretation of the results. All participant documentation for the BRIGHT trial, including the qualitative component, was developed through consultation with the youth forum.

A theoretical framework of acceptability (TFA) consisting of multiple domains, suggested by Sekhon et al. [13] was used to guide data analysis. The domains include: 1. affective attitude—how one feels about participating in the intervention; 2. perceived effectiveness- likelihood of intervention aim being achieved; 3. ethicality- fit with personal beliefs; 4. intervention coherence—understanding the intervention and how it works; 5. opportunity costs—what is given up in order to participate; 6. self-efficacy—confidence in performing the required actions; and 7. burden—the effort required to participate.

### Participants

Pupils, who had not explicitly withdrawn from the trial, were selected from the list of participants in the intervention arm of the BRIGHT trial and invited to participate. Pupils were identified from BRIGHT trial records by means of purposive maximum variation sampling using the variables of year group, gender, and regional location.

Members of school staff who were involved in the delivery of the lesson were invited to participate such as teachers, those in leadership roles such as the Head of Year and others such as the school nurse and learning managers.

The sample for all participants was drawn from the BRIGHT trial sites in England, Scotland and Wales and involved six secondary schools with above average proportion of pupils with free school meals. The sample for this qualitative study included 50 pupils (25 girls and 25 boys) aged 11–13 years and 12 members of school staff (9 females and 3 males).

### Recruitment and consent

#### Pupils

Schools distributed documentation about the BRIGHT qualitative study for pupils to take home. This included a participant information sheet, a reply slip and a parents’/carers’ cover letter to inform them that their child was being invited to participate in the qualitative study. The focus groups were then arranged through the school for those pupils who expressed an interest by returning the reply slip. Before beginning the focus group, the researcher obtained written consent from all participants. There were no drop-outs from those who had registered interest. Recruitment continued until no new themes were observed during the group discussions.

#### School staff

Schools distributed study documentation to potential staff participants. They were given the choice of having either a face-to-face or telephone interview. Those that registered an interest were then contacted to arrange an interview. Before beginning the interviews, the researcher obtained written consent for face-to-face interviews and verbal consent for telephone interviews (Additional files 1, 2).

### Data collection

#### Pupils

Six focus groups were conducted with 50 pupils (25 girls and 25 boys) aged 11–13 years in the intervention arm from six secondary schools across the UK (three England, two Wales, one Scotland). The focus groups took place at each school and were facilitated by experienced qualitative researchers (SE, HL, RJ, MR, ZM) with different academic backgrounds, including dentistry and social science. The moderators included four females and one male. Additionally, four of the focus groups were facilitated by two peer mentors, who were young people from the BRIGHT trial youth forum which was run by the charity ‘Children and Young People Empowerment project’ (Chilypep). The peer mentors were trained and supported by a Chilypep youth worker (EM) and a member of the research team (SE).

Before the focus group commenced the moderators introduced themselves and explained why they were interested in conducting this study. During the focus groups a member of school staff was present in the room. Prior to interviews, it was established that  there was no established relationship between the moderators and the participants. At the start of the focus groups an introductory activity was undertaken as an icebreaker.

The focus groups lasted on average 45 min (ranging from 35 to 55 min) and field notes were made after the interview and used to provide additional context to the analytical process.

#### School staff

Semi-structured interviews were conducted with 12 members of school staff Four interviews were face-to-face and conducted at school and eight via telephone. These included teachers (n = 6), learning managers (n = 2), and those in senior leadership roles (n = 4). Interviews lasted on average for 20 min (range 15–25 min).

The topic guides for use with pupils and staff were developed from the literature using the TFA and its operationalisation [13, 15]. The topic guides were kept flexible, allowing for the discussion of unanticipated issues and their incorporation into subsequent interviews.

Interviews continued with pupils and staff until data saturation was reached hence the sample size. All interviews were audio-recorded and subsequently transcribed verbatim and anonymised. All interview participants (pupils and staff) received a £10 Love2Shop voucher to thank them for participating. Data collection took place between June 2019 to November 2019.

### Data analysis

The software NVivo Version 12 QSR International was used for qualitative data handling; providing data management and retrieval facilities to support analysis and write-up. Data were analysed using the framework approach; a matrix-based method for the analysis of cross-sectional qualitative data designed to be rigorous and valid [16]. A pragmatic approach was adopted that drew on both deductive and inductive processes, enabling the exploration of a priori themes identified from the literature search and allowing new themes to be identified. The analysis involved the following stages: familiarisation, identifying initial themes, labelling the data, sorting the data by theme and synthesising the data.

Two experienced doctoral researchers (SE, HL) primarily analysed the data. This involved reading and re-reading the transcripts to achieve familiarisation with the data and independently identifying initial themes. SE and HL then independently and systematically coded transcripts and any discrepancies in coding were resolved through discussion. Additionally, any relevant field notes taken were used to help interpret the data. An a priori thematic coding framework was used to line-by-line code the transcripts and was developed from several sources: the TFA [13]; familiarisation with the interview transcripts; and research team discussion (SE, ZM, HL). Using the NVivo retrieval facilities, researchers remained connected to the original raw data throughout the refinement stages and the text could be revisited to verify conclusions. Further refinement was undertaken by SE and discussed with HL and ZM and any discrepancies discussed and resolved. This process strengthened inter-rater reliability and credibility and thus ensured the trustworthiness of the data analysis.

## Results

The findings presented below are based on the constructs of the TFA [13]. Five dimensions were identified, affective attitude, perceived effectiveness burden, ethicality, and self-efficacy. Several themes were identified under the dimensions of affective attitude and perceived effectiveness. Regarding the classroom-based session, some themes were present for both staff and pupils and some themes were exclusive to either staff or pupils. These are outlined in Table 1.

**Table 1:** Acceptability of the BRIGHT intervention components based on the theoretical framework of acceptability

Construct	Theme
**Class-room based lesson**	
Affective attitude and perceived effectiveness	*Engagement (staff and pupils)* *Materials and activities (staff and pupils)* *More information (pupils)* *Curriculum (staff)*
Self-efficacy	*Confidence in delivering the lesson (staff)*
Ethicality	*Importance of oral health (staff and pupils)*
Burden	*Preparation required (staff)*
**Text messages**	
Affective attitude and perceived effectiveness *(pupils)*	*Frequency and repetitiveness* *Timing* *More information* *Engagement* *Control*
Ethicality *(pupils)*	*Importance of oral health*

Throughout the results section, quotes are presented using the following nomenclature: For staff participants, school identification number and participant number are indicated in brackets e.g. (School staff 37:1) and for pupil participants, a focus group abbreviation followed by the school identification number and year group are indicated in brackets e.g. (Pupil FG: 57: 7), where year 7 includes 11–12 year old pupils and year 8 includes 12–13 year old pupils. Focus group participants are denoted as SX e.g., S9. Each pupil participant has a different number.

Overall, the intervention was described by pupil participants as interesting, helpful and informative:

*S9*: *Helpful… Totally informing*
*S8*: *Interesting*
*S7*: *Good *(Pupil FG: 75: 8)

Pupil participants reported that the lesson provided oral health knowledge and the texts reinforced the need for twice daily tooth brushing. Although the texts were described by some as ‘annoying’ they were perceived as useful brushing reminders.

*S3*: *The lessons help you understand. And they text you. So it gets in your head. They get annoying. Then you have to start doing that. That’s fun.* (Pupil FG: 16: S1)
*S5*: *Yeah. Now we’ve learned it, how to do it and like quite properly. *(Pupil FG: 57: 7)

Staff participants also found the intervention to be acceptable. Their involvement with the intervention was limited to the lesson component and they reported the lesson as *“all in all, it worked fairly well in terms of pupils, fairly easy to follow, easy to deliver”.* (School staff 78:1).

The TFA domains of opportunity costs and intervention coherence were not identified from the data. From the interviews conducted, participants did not mention missing out any opportunities due to their participation in the intervention. Similarly, no data was obtained that suggested the participants were aware of any coherence or indeed incoherence of the intervention; however, the participant's understanding of the mechanisms of action of the intervention was not directly probed during the interviews.

Further detail regarding the acceptability of the components of the intervention, text messages and the lesson, will be presented separately throughout the next section according to the TFA constructs outlined in Table1.

### Classroom based session: pupils and school staff

In line with the TFA [13] the dimensions identified for participants acceptability of the lesson included affective attitude, perceived effectiveness, self-efficacy, ethicality, and burden.

#### Affective attitude and perceived effectiveness

The data suggest that understanding the potential benefits of the lesson in improving oral health, affected the way participants felt about it. Due to some overlap in the dimensions of affective attitude and perceived effectiveness they have been presented jointly. Overall, both staff and pupils found the lesson acceptable. Staff described the lesson as a success and reported that it had gone well. They described delivery as “*an enjoyable experience”* (School staff 37:1).

*It was good. It was very thorough. I would say that it was a success…Yes, for year sevens and eights, I would say it was appropriate definitely.* (School staff 33:1)

For the most part pupils found the lesson helpful and it led to some participants reporting being more interested in oral health as a result. In particular, understanding the consequences of poor oral hygiene prompted pupils to reflect on their oral hygiene and the importance of brushing their teeth.

S7: *I didn’t know you had to brush in two minutes so I used to do it a minute but now I do it for two minutes.* (Pupil FG: 37: 7)
*S7*: *Like what would happen if I didn't brush my teeth every day and I didn’t used to be like interested in that before.* (Pupil FG: 75: 8)

Pupils reported the lesson *“was covered well”* (Pupil FG: 37:7) and they found it interesting and fun. They valued the use of the lesson to inform them more about oral health, stating that it *“made you think”* (Pupil FG: 16: S1). However, for some pupils the lesson was a bit long, and they found they had become bored and disengaged by the end.

*S3*: *They’ve done it in a way that it was interesting like fun. But then, like it wouldn’t be too boring, but like you’re learning about something that you don’t know you want to learn about. But then when you’re doing it, you actually enjoy doing it.* (Pupil FG: 16: S1)
*S6*: *At the end it got very boring….because it’d been like really long*. (Pupil FG: 57: 7).

##### Engagement-staff and pupils

Some staff participants reported that the content of the lesson was appropriate and consequently pupils “*were very engaged with it”* (School staff 33:1) however, others reported that the content was not engaging enough for some of their pupils.

*I think it's probably the areas as well and it'll be different levels of abilities in different areas and different, I mean, the area that we're based in is more of a deprived area. So actually, think is probably…it was probably better to be at that level for the students.* (School staff 33:1)

*….well we have boys…that play on quite high-level computer games and things like that. It wasn’t gripping enough for them* (School staff 39:1)

*And, I think it needed to be—slightly more interactive, so we tried to find a way of making it more interactive and a bit more so they could participate a little bit more.* (School staff 38:1)

The setting of the delivery was also important. Those who received the lesson as part of a whole year assembly reported that they did not get the opportunity to ask questions, either because they did not get the chance or because they did not feel like asking questions in front of such a large group.

*S6*: *I didn't really get the chance to ask why we should brush our teeth twice a day so yeah.*
*S7*: *And I didn't feel like I could ask questions because there was so many people. *(Pupil FG: 75: 8)

##### Materials and activities- school staff and pupils

Overall, staff found the materials and activities provided for the lesson to be suitable and appropriate. There were different views however, on whether the resources and lesson plan were appropriate for the duration of the lesson. Some described it as being suitable for the time allocated whereas others felt there was too much content for the time allocated. On the other hand some staff stated that it was difficult to make the lesson last for an hour or 50 min due to insufficient content and that they were “*going slow towards the end to you know, to create an hours lesson whereas it was probably more of a 35, 40 min lesson.”* (School staff 62:1).

*…..our lessons are 50 minutes long. Now that was a stretch to keep that going for 50 minutes. ….. so there wasn’t enough content to keep* (School staff 39:1)

*I think the resources that were given were enough. They fitted into the presentation well. There was a video as well. It was absolutely fine. I think it was the right kind of as well….I think with the activities that the students had to do with the time that you spent discussing things as well as obviously, doing the presentation and talking about it, it was a nice mixture. And it, I think. it lasted in total about an hour. So it was a good length, I think, to engage the students.* (School staff 33:1)

*S7*: * Like it was like telling me like how to brush your teeth and stuff like that. It was like…it just felt like it wasn't meant for our age. *(Pupil FG: 33: 8)

For some members of staff one element of the lesson plan (an educational animated video on tooth brushing) was considered “*babyish for the year group that it was targeted at”* (School staff 75:1). Indeed, a number of pupils also echoed the view that the video was too childish for them. The pupils interviewed stated that they already knew how to brush their teeth, the video was not seen as useful, instead they would have preferred to understand more about why tooth brushing is important.

*S7*: *Like it was like telling me like how to brush your teeth and stuff like that. It was like…it just felt like it wasn't meant for our age. *(Pupil FG: 33: 8)

Both pupils and staff spoke positively of the photos that were provided as part of the lesson to illustrate the consequences of inadequate tooth brushing and the pupils enjoyed learning more about oral health.

*Moderator What were your favourite parts?*

*S6*: *I enjoyed it …yeah and when they showed us the pictures of really disgusting teeth.*
*S11*: *And they talked about facts and stuff, that I found interesting*. (Pupil FG: 75: 8)

*I liked the pictures that you sent of the before and after sort of pictures that you sent, and they liked those as well.* (School staff 39:1)

##### More information-pupils

When asked about how the oral health lesson could be improved, more information on oral health was suggested by some:

*S6*: *Why brush your teeth twice a day*
*S7*: *How different toothpastes like affect your teeth and how they help them.*
*S8*: *And what mouthwash to use as well.* (Pupil FG: 75: 8)

Pupils also suggested more visual material such as photos and videos for providing information on oral health. They requested more information about what could happen if they didn’t brush their teeth and felt that graphic pictures of this could be powerful in influencing their behaviour.

*S13*: *And like show more like stuff what could happen, you know, like more graphic pictures. It’s like what they do with the car crash isn't it like showing like everywhere was like learning, like great graphic images so then it’s stopping dangerous driving.* (FG: 33: 8)
*S7*: *There could have been more videos.*
*S4*: *More videos.*
*S7*: *Yeah, and less like talking about it.* (FG: 75: 8)

There was also the suggestion of several lessons over the pupil’s time at secondary school, rather than relying on a one-off lesson.

*S7*: …..*more lessons would be really helpful to tell us more about like teeth. *(Pupil FG: 75: 8)

##### Curriculum-staff

Introducing new content for schools to cover can be challenging particularly when it does not relate to national qualifications. The incorporation of the content covered by the BRIGHT classroom-based lesson into the PSHE curriculum was felt to be a positive factor contributing to the acceptability of the lesson on oral health.

*……but now obviously, with the government agenda which is to prepare students to…… they’ve introduced PSHE now……so, it’s gone into the curriculum, so that, will allow far more flexibility to put that in because it ticks a lot of boxes for PSHE.* (School staff 38:1)

#### Self-efficacy

##### Confidence in delivering the lesson- school staff

When probed about the acceptability of the lesson, members of staff in leadership teams raised the importance for those delivering a lesson on oral health to feel confident teaching the subject area by having sufficient knowledge and support.

*Probably that lack of knowledge themselves maybe, lack of confidence in delivering it if they didn’t have that knowledge.* (School staff 62:1)

*It's something that we’d maybe have to look at freeing up so that maybe she could have a conference call with somebody beforehand to go through it all, yeah, and just maybe just be given a little bit more at our end as well to be able to give her the confidence in delivering it.* (School staff 75:2)

#### Ethicality

##### Importance of oral health- school staff and pupils

Furthermore, the acceptability of the intervention was also attributed to the personal beliefs and values of the staff and perceiving it as “*worthwhile”* (School staff 39:2). Those who valued the importance of oral health and recognised the detrimental impact of poor oral health on young people’s well-being were more likely to appreciate the oral health intervention.

*I thought it was a good idea because we’ve got a lot of kids losing their teeth so I felt it was definitely worthwhile.…one of the girls who was in the class told me… she was, what, 12, and she’d already had 8 teeth out, so that made it feel like this feels important.* (School staff 37:1)

In turn, personal beliefs of oral health as part of general health underpinned staff members’ positive interpretation of curriculum objectives such as promoting personal hygiene.

*…..if we put it straight into that personal hygiene sort of framework, how you keep yourself healthy in all aspects….So, it’s about your body health, your mental health, your physical health…..So, I think it all ties straight into that.* (School staff 38:1)

#### Burden

##### Preparation required- school staff

Another important aspect of the acceptability of the lesson from the perspective of staff members was whether they would be required to dedicate time and effort into preparing it. Those delivering the lesson were relieved when they were made aware that they were not required to prepare anything for the lesson and mostly appreciated the resources that had been developed.

*S2*: * They said that the resources were good, they were glad to have everything to hand...they were grateful they didn’t have to prepare anything, …….they were happy to get on board with it. It’s no problem at all, I think I may have had some initial comments about whether or not they have to prepare the resources and once I assured them that that was taken care of and they just have to you know, review the material before the lesson, and then deliver it, they were happy with that.* (School staff 62:1)

However, having to print off the materials and arranging a dedicated time for the lesson was seen as a burden.

…*.that someone just has it dropped on them as an additional extra like what I was given…obviously the printing as well, obviously that took money out of my budget that wasn't necessarily signposted for this* (School staff 75:1)

Moreover, this particular school did not have dedicated PHSE lessons, which made it logistically challenging in finding the time to incorporate something new.

*We don’t have PSHE lessons. No, we teach that throughout the curriculum. So that's what I’m—like the logistics of getting them all together ….And it did work well but like I said, it did take a lot of teacher time for me to prepare it and make sure it was ready.* (School staff 75:1)

### Text messages

#### Affective attitude and perceived effectiveness

Overall, pupils reported that the text messages were useful reminders to brush their teeth. Participants generally felt the language used within the texts was appropriate however, some described them as *“cringey”* (Pupil FG: 37: 7) and over time the texts became *“annoying”* (Pupil FG: 75: 8).

*S6*: *The language is just perfectly fine for our age group.* (Pupil FG: 62: 8)
*S5*: *I think the most helpful thing ….was like the reminding that like help me do that* (Pupil FG: 16:S1)
*S6*: *I find it nice and good because it’s just saying about like teeth, like “Have you brushed your teeth?” …….and then like it makes you think, “Oh yeah” and then I’ll go through my teeth. It’s definitely better than just, someone in your house still telling you to do your teeth like it’s better than them—like it’s more…. like pleasant.* (Pupil FG: 57: 7)

Some of the students reported that the texts had brought about positive changes in their habits as they helped them to brush their teeth twice a day- something that they did not usually do. For others the texts were perceived as effective as they felt they were benefitting from them in terms of improved oral health: “*it’s even better that we’re getting something from it and good teeth”* (Pupil FG: 57: 7). The perceived effectiveness of the text messages can also be evidenced in the fact that even those who found the text messages annoying still found them helpful enough to choose to continue receiving them.

*S15*: *It was good but I found that the text messages were really annoying…… I do like to use it as a reminder to brush your teeth.* (Pupil FG: 33: 8)
*S11*: *It was a nice like reminder to remind me about brushing my teeth every day anyway.*
*S7*: *I’m not used to brushing twice a day, but it helped me to brush them twice a day*
*S6*: *They helped me remember in the night because I didn't use to do it in the night but I do now. *(Pupil FG: 75: 8)

##### Frequency and repetitiveness

Pupils attributed the frequency and repetitiveness of the text messages as two reasons why they found them annoying. Participants felt that the texts came too often and were bored of how often the same message was repeated. This may have led to the pupils disengaging with the oral health messages in the texts as the frequency and repetitiveness was reported to *“drains your energy after a while”* (FG: 33: 8). The pupils felt that more varied and creative messages would improve the texts and make them more interesting.

*S6*: *Got them every single day.*
*S10*: *Like how often they come, like not so much.* (Pupil FG: 75: 8)
*S8*: *Just like they're helpful, but they’re a bit like repetitive and like they don't vary, is like a set five that just keeps repeating.* (Pupil FG: 33: 8)
*S6*: *Maybe it should be creative, more creative with texts. (Pupil FG: 16: S1).*

Despite being described as annoying, participants still found the texts to be helpful reminders that prompted them to “*get up and brush your teeth”* (Pupil FG: 57: 7).

*S7*: *I got frustrated there, I kept if for a few weeks and then I blocked it after.*
*S10*: *I blocked it because I know what times to do them, I just blocked it because I knew my routine.* (Pupil FG: 37: 7)

For some pupils however, the perceived tediousness of the texts eventually outweighed their usefulness and they reported blocking the messages as they had “*done my head in”* (Pupil FG: 62: 8) while others reported blocking them as they felt confident regarding their tooth brushing routine.

Furthermore, some participants spoke of their disappointment upon receiving a text message and realising it was from the BRIGHT research team rather than from one of their friends.

*S7*: *Makes you feel important like somebody’s trying to talk to you and then it’s like, no.* (Pupil FG: 16: S1)

Additionally, being interrupted during mobile phone activities such as playing games was mentioned by some pupils as to why they were annoyed by the texts.

*S4*: *When I’m playing something or like watching something it just pops up and the game just freezes when I’m like about to win so it gets me annoyed a bit. The way I can say to make it a bit better is if we were just like a notification, just like it went ding or something like that*
*S5*: *It just keeps popping up when you’re like playing stuff.* (Pupil FG: 57: 7).

##### Timing

Participants were given a choice regarding the delivery times of the text messages. There were two set times to choose from for receiving the morning texts and two set times for the evening texts. The time slots offered on weekdays differed to those offered on weekends with weekends having later delivery times in the day. Overall, pupils reported that the timings of the SMS delivery on weekdays were suitable however some would have preferred different times to suit their personal circumstances.

*S11*: *I think the timing was suitable for my timetable. Like I wake up a couple of minutes before got the message and then the message came and then that would usually be the time I'd go down and brush my teeth*. (Pupil FG: 75: 8)
S2: *No, because I play football. I don’t get home sometimes till half nine.* (Pupil FG: 16: S1)

More choice of SMS delivery times was recommended particularly on weekends and holidays where teenagers were more likely to sleep in and stay up late.

*S11*: *I think on weekends and holidays, they could have been a bit later…...Like I wake up around 11:00*. (Pupil FG: 75: 8)

##### More information, engagement and control

Another recommendation as a way of improving the text messages was to provide more information regarding oral health. Pupils spoke of their preference of informative texts offering more facts. Additionally, being able to interact and engage with the content delivered was important to pupils. They expressed their preference of being active receivers of information. They suggested an app which would allow them more control in setting the delivery times of the reminders while also serving as a resource of information with more detailed content and videos that they could access for information.

*S4*: *Like an interactive app…Like you can do stuff.*
*S3*: *So like you can have an app with how many times you should brush your teeth regularly, and then facts about it, and then like your reminder, something like that.*
*S4*: *Yeah, so maybe some of the things you had in there…in the lesson be in the apps that you could go back to it.* (Pupil FG: 75: 8).

#### Ethicality

##### Importance of oral health

Pupils appreciated the messages and identified them as *“good”* (Pupil FG: 57: 7) based on their own personal beliefs of oral health. For some participants their own personal beliefs and values of oral health led them to restart receiving text messages despite initially being angry and stopping them.

*S4*: *Well it’s good that like they want to encourage young people to like brush their teeth. So yeah, it just…. I mean like it influences them not to have like teeth like smoker’s teeth or teeth that people don’t brush properly and they’re all just like holey and yellow and black.* (Pupil FG: 57: 7)

*Moderator Does anyone block the messages?*

*S5*: *At one point I was because I got really angry with it.*

*Moderator And then what made you start up again?*

*S5*: *But it’s like something good because it reminds you to brush your teeth.* (Pupil FG: 57: 7)

## Discussion

This study aimed to assess the acceptability of the BRIGHT intervention components; classroom-based lesson and text messages from the perspective of pupils and school staff members.

Overall, both participant groups found the intervention to be acceptable and pupils described the text messages as useful reminders for brushing. This is in line with the findings of other studies which have reported on the acceptability of text message behaviour change interventions for young people. These have included interventions aimed at improving clinic attendance, oral hygiene, physical activity and weight management, contraception use, sun-protective measures or reducing smoking and alcohol misuse [17–19].

The findings of this study demonstrate that staff recognised the value of the lesson and reported that in general the content was suitable for their pupils. Having the lesson material prepared for them, having the nepessary support and  the requirement for it to be included in the curriculum were factors that improved acceptability. Teachers appreciated having prepared material as it was one less task to undertake however they also found it important to have some flexibility in delivering the lesson. Flexibility was needed to adapt the lesson appropriately to suit the school context such as the duration of lessons and different abilities within a particular cohort of pupils. Additionally, it is important to point out that recent guidance [20, 21] now requires oral health to be included as part of the curriculum however at the time of intervention this was not the case. Nonetheless, some school staff members were aware of the upcoming changes and this appears to have improved the acceptability of the lesson. Pupils also spoke positively of the lesson and particularly appreciated the visual material. Pupils recommended providing more oral health information and adding more than one lesson to reinforce what they had learned.

Recently, oral health has become a compulsory requirement in England [20] and Scotland [21] meaning it will now be embedded within the formal curriculum, instead of being an optional add-on. This will ensure it is provided as part of pupils learning. This is significant in terms of improving young people’s oral health. Nonetheless, it is important to remember that the formal curriculum is only one of the three interacting spheres of a health-promoting school as described by the WHO’s Health Promoting Schools framework. Moreover, it is vital that the necessary structures are in place, such as conducive healthy environments, to enable pupils to apply their knowledge as education alone is insufficient to support behavioural change.

The text messages were developed rigorously and co-designed with pupils during the intervention development stage. This approach was taken based on the recommendation of previous studies to ensure that the texts were appropriately written for the target population, tailored according to their age and used the participant’s name. The findings of this study suggest that in general they were acceptable. Some pupils however, found the texts “annoying” and described getting fed up with them due to their frequency and repetitiveness. A schedule of 28 different messages was repeated every fortnight. Some pupils also described them as “cringey” due to the wording. Consequently, some pupils became frustrated with the texts and reported blocking or muting them. While the text messages were designed with young people’s involvement and a short pilot was undertaken, this finding shows the importance of gaining the views of those who have experienced receiving the intervention over time. This is significant in terms of intervention effectiveness as participant engagement needs to be maintained. Boredom, annoyance, habituation (ignoring messages) and alert fatigue have been reported pitfalls of mhealth interventions that potentially affect long term engagement [22–24].

The BRIGHT intervention provided the option for pupils to request the messages to stop being delivered to their number by texting back STOP. However, pupils only spoke of blocking or muting them rather than going through the formal mechanism of the trial for stopping delivery of the messages. As part of the wider process evaluation, one of the variables being measured is the number of pupils that the texts are being delivered to in order to assess fidelity. The focus group interviews revealed that some pupils blocked or muted the texts and therefore the texts were still delivered to them but in effect were not received or read. It was not possible however to record how many messages were blocked or muted. Indeed, some pupils appeared to have self-regulated taking some time off from receiving the texts. As they reported blocking the messages and subsequently unblocking them when they realised the importance of the texts for improving their oral health. When evaluating the mechanisms of impact of an intervention acceptability is a key factor and the findings of this study suggest that in general it was acceptable to both staff and pupils. Additionally, the dose of the intervention received is also an important factor to consider. The findings suggest that the actual dose of text messages received by pupils in effect does not equate to the dose of text messages sent.

In addition to adding extra insights, the pupil interviews provided recommendations for improving the acceptability of the texts. These included more choice over delivery times, more information and more interactivity. Some pupils suggested an app may be better than text messages. They described the ideal app as one that could be customised to fit their own personal routines, served as an informative resource and sends reminder notifications to brush their teeth. While texts do lack the interactivity of an app, relying on an app alone is not without its’ limitations. Reminders would still need to be sent twice daily, in accordance with brushing guidelines, and thus the frequency and repetitiveness may still lead to young people finding them annoying. The use of an app (rather than text messages) may exclude those children without smartphones, those with insufficient space/storage/data on their phone to install the app or those with older models that may be incompatible with the app or unable to make the necessary software updates [25, 26]. There is evidence that suggests that oral health interventions that used a mixed approach, including text messages and an app, were more effective than using either approach alone [19].

Future interventions should consider having a more varied SMS schedule, piloting the texts with a youth forum for a longer period of time, and delivering the lesson plan to a class of pupils rather than an assembly. The findings of this study also have implications for policy change in that they support the incorporation of oral health into the curriculum throughout primary and secondary education.

All efforts were made to minimise the power imbalance between the researchers and pupils, including having young people from the youth forum facilitate four of the focus groups, using a familiar venue, and starting the focus groups with an ice-breaking introductory activity. Additionally, the study was designed with input from a multi-disciplinary research team with experience of conducting interviews with children and young people.

A limitation of this study was not being able to explore further why some participants asked to stop receiving the texts, after receiving them for several weeks. This was due to maintaining the anonymity of participants therefore they could not be purposively sampled. It is likely that the reasons that have been reported for blocking or muting the texts are most probably the same for those that requested to stop receiving the text messages. The quantitative analysis of the wider process evaluation at the conclusion of the trial, will provide more information regarding the number of participants who requested to stop receiving the texts and then requested to receive them again however the number of pupils who blocked or muted the messages cannot be assessed. It is acknowledged that the thank you voucher, given to the participants for their time taking part in the interview, may potentially have led to response bias from some participants. However overall, the data captured both what participants liked and disliked about the intervention.

## Conclusion

Overall, pupils and staff members found the lesson and text message components of the BRIGHT intervention acceptable. Oral health education being embedded within the school curriculum played a significant role in improving acceptability for those delivering it. Future research should consider the recommendations made participants to improve acceptability of the components which included more choice over delivery times, more information, and more interactivity.

## Supplementary Information

**Additional file 1**. BRIGHT Focus Group Topic Guide- Pupils.

**Additional file 2**. BRIGHT Interview Topic Guide- School staff.

**Additional file 3**. Consolidated criteria for reporting qualitative studies (COREQ): 32-item checklist.

## Data Availability

The datasets analysed in the current study are available from the corresponding author on reasonable request.

## Abbreviations

BRIGHT: Brushing RemInder 4 Good oral HealTh
SMS: Short-message service
PSHE: Personal, social, health and economic education
TFA: Theoretical framework of acceptability
FG: Focus group
