# Supplementary material for: Acceptability of the Brushing RemInder 4 Good oral HealTh (BRIGHT) trial intervention: a qualitative study of perspectives of young people and school staff
Source: BMC Oral Health. 2022 Feb 23;22:44. doi: 10.1186/s12903-022-02073-w (PMC8864777; doi:10.1186/s12903-022-02073-w)
Supplement: Supplementary file 1 — Additional file 1. BRIGHT Focus Group Topic Guide- Pupils. [file 12903_2022_2073_MOESM1_ESM.docx]

**Additional File 1. BRIGHT Focus Group Topic Guide- Pupils**

*Flexibility should be used when undertaking the interviews and applying the topic guide in terms of wording of questions, order of questions, use of probes/prompts and every opportunity made to allow participants to raise their own issues.*

**Opening:**

Introductions:

- Interviewer to introduce themselves and thank them for their participation.

**Establishment of ground rules**

- No right or wrong answers we just want to find out what you think
- Confidentiality – information collected during the study is confidential and access will be restricted to our research team. Some of your comments may be included in a report on the study or in articles for scientific journals but these will not use your real name. Please don’t repeat what other people say outside of this session.
- To help us with this study, we are going to use this recorder to make a recording of what we all say today. Nobody will be able to identify you from that recording other than me.
- Everyone’s views are of interest
- Aim to hear as many different thoughts as possible
- Likely to be different views, feel free to say what you think – OK to agree/disagree with others
- Don’t wait to be invited before stepping in, but please don’t talk over each other
- Might make some notes while you’re speaking – just to jog memory
- Any questions?
- Check consent forms of participants

**TURN ON AUDIO RECORDER and inform participants the recorder is now on.**

**Dental health lesson**

- Who delivered the dental health lesson?
- How was it delivered? Probe: lesson, an assembly, a combination of both or something else
- Was it delivered as part of PHSE or Life lessons?
- Would you have preferred it to be delivered differently? Why?
- Do you think the content of the lesson was pitched at an appropriate level for you?
- What did you think of the duration of the lesson? Was it too short or too long?
- What did you think of the timing of the lesson? Probe: early morning, mid-day, late afternoon or before break/lunch or early in the week on a Monday vs an afternoon lesson on a Friday
- Did the lesson stay on topic? If not, why?
- Did you think the lesson was engaging? If so, why?
- Did you think the lesson was boring? If so, why?
- Did you watch the video clip?
- What did you think of the video clip?
- What did you think of making the toothbrushing plan? Probe: was it useful? Have you used it since?
- Did you receive and read the factsheet?
- What did you think of the factsheet?
- Was there anything in the lesson that you remember finding a bit difficult to understand or wasn’t clear?
- If so, were you able to ask the teacher questions?
- If so, were they able to answer your questions?
- Did you think that the teacher was well-prepared for the lesson?
- What did you like or not like about the lesson?
- Do you think it could have been delivered better? How?

**Text messages**

- What do you think of the content of the text messages? Probe: were they boring? Cringey?
- Do you think they are appropriate for your age group?
- Do you think the timing the text messages were sent was appropriate for you? Probe: during the week? At weekends? In school holidays?
- Did you receive the text messages on your own personal phone or your parents phone?
- When the text messages were sent did you read them? Probe: did you ignore, mute or block them?
- Are you still receiving the text messages? Have you texted back STOP? Why?
- How did you feel when you received the text messages? Probe: did they encourage you to brush your teeth, or did you get bored/annoyed after receiving them for a while?
- What did you like or not like about the text messages?
- Do you know what to do if you change your phone number?
- What do you think could have improved the text messages?

**Intervention overall**

- Do you feel that attending the lesson on oral health and receiving the text messages has helped you to brush your teeth?
  - Why? Probe: what has helped the most? Do you feel it has changed your attitude towards brushing your teeth?
  - Why not? Probe: Were there any barriers that prevented you from brushing your teeth?

**Closing**

- Is there anything you would like to add, anything we’ve missed out?

[Switch off recorder]

Participants will be thanked for the discussion and given gift voucher. Participants will be de-briefed on the next steps of the research process.
