# Supplementary material for: Acceptability of the Brushing RemInder 4 Good oral HealTh (BRIGHT) trial intervention: a qualitative study of perspectives of young people and school staff
Source: BMC Oral Health. 2022 Feb 23;22:44. doi: 10.1186/s12903-022-02073-w (PMC8864777; doi:10.1186/s12903-022-02073-w)
Supplement: Supplementary file 2 — Additional file 2. BRIGHT Interview Topic Guide- School staff. [file 12903_2022_2073_MOESM2_ESM.docx]

**Additional File 2. BRIGHT Interview Topic Guide-School staff**

*Flexibility should be used when undertaking the interviews and applying the topic guide in terms of wording of questions, order of questions, use of probes/prompts and every opportunity made to allow participants to raise their own issues. Questions will vary depending on different staff roles.*

**Opening:**

Introductions:

- Interviewer to introduce themselves and thank them for their participation.

**Establishment of ground rules**

- No right or wrong answers we just want to find out what you think
- Confidentiality – information collected during the study is confidential and access will be restricted to our research team. Some of your comments may be included in a report on the study or in articles for scientific journals but these will not use your real name.
- The conversation will be recorded however nobody will be able to identify you from that recording other than me.
- Any questions?
- Ask them to sign consent form if one has not been sent in.

**TURN ON AUDIO RECORDER and inform participant the recorder is now on.**

Health topics in the curriculum

- Has poor dental health been something your school has been concerned about in the past?
- Did you previously cover health topics in the curriculum such as dental health including tooth brushing, visiting the dentist?

**Dental health lesson**

- Who delivered the dental health lesson?

Probe: Specialised PHSE teachers or teachers used to teaching other disciplines?

- How was it delivered?

Probe: lesson, an assembly, a combination of both or something else

- Was it delivered as part of the school’s PHSE curriculum or some other part of the programme?
- When was the BRIGHT lesson delivered? date(s)
- Do you think the content of the lesson was pitched at an appropriate level for the age group?
- What did you think of the duration of the lesson? Was it too short or too long?
- What did you think of the timing of the lesson?

Probe: early morning, mid-day, late afternoon or before break/lunch or early in the week on a Monday vs an afternoon lesson on a Friday

- Did you think that the students found the lesson engaging?

Probe: Were there any signs of engagement in any of the different activities?

- Did you think that the students found the lesson boring?

Probe: Were there any signs of lack of student engagement in any of the different activities?

- Did the lesson stay on topic? If not, why?
- How did the lesson plan go?

Probe: Were you able to include the video clip, photos, factsheet, booklet, toothbrushing plan?

- Was there anything particular in the lesson plan that you remember the students finding it difficult to understand?
- Did students ask any questions? Were teaching staff able to answer these?
- Were there any adaptations you felt you had to make to the original lesson plan?
- Now that you have given the lesson, is there anything you think could improve it?

**Text messages**

- When the students started getting their text messages did they ask any questions about them or mention them at all?

Probe: students forgetting they had signed up to receive text messages as part of BRIGHT

**Intervention overall**

- Have the students mentioned any changes to their tooth-brushing behaviour as a result of the dental lesson and receiving the text messages?
- Are you aware of any other school-based activities this school has taken part in to improve dental health? Healthy school programme? Water in schools initiatives? Etc

**Closing**

- Is there anything you would like to add, anything we’ve missed out?

[Switch off recorder]

Participants will be thanked for the discussion and given a gift voucher (for telephone interviewees the voucher will be sent to the school).

Participants will be de-briefed on the next steps of the research process.
